# Innovation in Neighborhood Management Web Service: A Precise Initiative to Augment Audiences' Interaction on Social Media

**DOI:** 10.3389/fpsyg.2022.920112

**Published:** 2022-07-07

**Authors:** Muhammad Waqas Sadiq, Chunhui Huo, Abeer S. Almogren, Norah Abdullah Aljammaz, Waleed Mugahed Al-Rahmi, Qusay Al-maatuok, Salman Zulfiqar

**Affiliations:** ^1^Business School, Liaoning University, Shenyang, China; ^2^Department of Management Sciences, Commission on Science and Technology for Sustainable Development in the South (COMSATS) University Islamabad, Sahiwal Campus, Sahiwal, Pakistan; ^3^Department of Art Education, College of Education, King Saud University, Riyadh, Saudi Arabia; ^4^Faculty of Social Sciences and Humanities, School of Education, University Teknologi Malaysia, Skudai, Malaysia; ^5^School of Digital, Technologies and Arts, Staffordshire University, Stoke-on-Trent, United Kingdom

**Keywords:** service innovation, social media, neighborhood management, online relationship quality, trust, satisfaction, online loyalty, online interaction

## Abstract

In this article, two significant elements in social media websites, system operation, and social technology are examined in connection to website visitors' online loyalty and interaction, namely, commitment and satisfaction, in neighborhood management through social media websites. A total of 287 social media users completed a systematic questionnaire. After confirmatory factor analysis, data were examined in AMOS 24 using structural equation modeling with bootstrap. The research showed that both variables indirectly influence website visitors' online loyalty and interaction *via* trust and satisfaction, but not directly. Online relationship quality characteristics impact the interaction pattern of social media users after changes in services applied by the respective organizations on their websites. As for managers, the research gives crucial data on user behavior in connection to new services launched by organizations on their websites and shows how value creation to the target audience may help them reduce costs and optimize revenues.

## Introduction

Neighborhood management and community governance could be considered as a pivotal issue for any government or city management body. Governments are trying their level best to provide top-tier facilities to their citizens in developed cities and communities and less-developed areas. For this purpose, they are constantly trying their best to develop new methodologies and resources to cope with such issues (Vogel et al., 2021).

The question is that how the organizations and governments could understand the mechanism and usage of social media and blogging to improve the feedback procedure from the target audience. That is why this research explores this critical topic to provide sustainable and emerging solutions relevant to society's modern community and municipal needs. With the rapid advancement in technology, it is made possible for the government and different organizations to tackle issues related to neighborhood management effectively and efficiently (Aleshire, 1971; Nakano and Washizu, 2021).

This is also extremely important for social media websites. If they want to collect data of their target respondents and analyse their responses from a neighborhood management perspective, they must provide innovative services to their final consumers. This action will help them to attract more users to their social media network hence more accurate data gathering related to their neighborhood management (Ghaderi et al., 2016; Monirifar et al., 2021).

To understand the concept of innovation, we need to start it from the very start, including innovation in products. Product innovation is an old notion that is used to improve a product's performance and appearance, as well as to attract and keep consumers. Innovation in services is a way to provide new ways of services with the help of your customer's feedback (Barras, 1986), and presentation of related services and thus provide new and improved service to achieve a higher level of customer satisfaction (Go Jefferies et al., 2021). Following this, Miles (1993) clarified the notion by stating that innovation in services may also result in process, management, people, or organizational restructuring to increase customer experience and satisfaction. Fruja and Jivan (2006) described this idea as a collection of various actions performed by the service provider in response to the client's request to understand its demands. With the development of new and inventive approaches and strategies (Dunleavy et al., 2021), social media websites need to provide and embrace new processes and technology. These may eventually result in changes to their services to better understand the requirements and desires of their target clients; as a result, they may stay loyal to them in the long term (Papastathopoulou and Hultink, 2012).

To accomplish this, Yang et al. (2014) conducted an exhaustive study on the construction of precise dimensions against which they could quantify the improvements in services given by various blogging platforms from the user's perspective (Akram et al., 2018b). They dubbed their new scale “BLOG—S—INNO,” consisting of five dimensions and 18 components. The aspects are an innovation in system operations, innovation in service privacy, innovation in web page content, innovation in social technology, and innovation in diversity (Al-Rahmi et al., 2021). These five dimensions give an excellent summary of the invention of blogging-related services. Blogging is an online activity that enables bloggers to share their contact information, thoughts, videos, text, photos, and links to other websites and sources of information, and videos (Allcott et al., 2019). By blogging, anybody may rapidly and effectively market their ideas, goods, and services, or anything else (Li and Chen, 2009; Shareef et al., 2019). Anyone may debate and give knowledge on any subject *via* blogging. Thus, it is an extremely beneficial and practical method of earning; moreover, by delivering relevant and trending content to visitors and target clients, individuals earn a respectable sum of money *via* blogging (Lou and Yuan, 2019).

More traffic to your website equals more revenue, which is why social media websites are anxious about their respondents and looking for efficient strategies to increase their respondents' loyalty to their website (Willment, 2020). Facebook is also regarded as a blogging platform since it enables users to easily and properly exchange contact information, text, photographs, and videos with others, and visitors may stay current on topics of interest (Yang et al., 2014; Chen et al., 2020). As a result, users gravitate toward various types of blogging websites to get information about desired goods or services and have a better understanding of associated services and products (Al-Rahmi et al., 2015; Al-Maatouk et al., 2020). Obtaining information from numerous blogging services assists one in making an informed choice about the usage or purchase of any product (Hansen et al., 2010). It has become vital to include innovation and creativity in the services supplied. Product innovation is a time-honored practice of enhancing products' performance, appearance, and customer retention. While Barras (1986) describes innovation in services as “new and improved ways of producing and offering services to customers to enhance the performance and presentation of connected services for the firm to achieve a higher level of customer satisfaction.” Miles (1993) elaborates on the concept, stating that service innovation may result in process, management, people, or organizational restructuring to boost customer satisfaction. According to Fruja and Jivan (2006), this concept entails a sequence of measures conducted by the service provider to get a deeper understanding of the client's requirements. The same is true for blogging services worldwide. Blogging is an Internet-based second-generation activity that is extremely popular these days for its effective communication and domino effect regarding different services (Ip and Wagner, 2008). Johnson and Kaye (2004) said that blogging is a web-based diary-style activity that provides observations, recommendations, and suggestions regarding different products and services in combination with commentaries and recommended links for different useful articles and videos. Li and Chen (2009) further clarify the concept of blogging by saying that blogs serve as a model of an online social network, which represents frequently updated web-based commentaries with reverse sequences of dated entries, which could be checked and read by the blog browsers at any time.

With the emergence of novel and innovative methods and techniques, suppliers of blog services must now embrace new processes and technologies (Den Hertog et al., 2010). These may ultimately result in improvements to their offerings to better understand their target clients' needs and objectives, hence retaining their loyalty (Papastathopoulou and Hultink, 2012).

To do this, Yang et al. (2014) undertook an in-depth investigation of the specific parameters on which consumers may assess advancements in blogging services. “BLOG—S—INNO,” or blog service innovations, is a five-dimensional object with 18 components. System operations, service privacy, online content, social technologies, and diversity are all considered. These five elements provide an excellent overview of the blogging service innovation process. Bloggers use the Internet to share their contact information, ideas, videos, text, photographs, and links to other websites, information sources, and videos (Allcott et al., 2019). Through blogging, anybody may quickly and efficiently promote their ideas, products, and services (Li and Chen, 2009; Shareef et al., 2019). Anyone may write about and debate any issue. Individuals may earn a substantial income from blogging by providing relevant and fashionable material to their visitors and target consumers (Lou and Yuan, 2019).

Increased traffic equals increased money, which is why bloggers are continuously concerned about their visitors or customers and searching for ways to boost client loyalty (Willment, 2020). For instance, Facebook users may readily communicate contact information, text, images, and videos with one another, while visitors can stay current on topics of interest (Yang et al., 2014; Chen et al., 2020). As a result, visitors visit a variety of blogging websites to discover more about the products or services they are interested in. Getting information from numerous blogging services is useful when deciding to use or buy a product (Hansen et al., 2010) by different organizations for their survival in this fast-paced emerging and technological economy to sustain their business (Liao et al., 2011). In Pakistan, Facebook is very active and popular, and in Saudi Arabia, Twitter is very active and popular. Facebook surpassed all other social media applications by having 30 million users, and as we have already discussed, Facebook is also considered a blogging platform. It is a useful and powerful platform to give new information and advice related to any topic. Bloggers are trying to keep up with the latest trends in their area of expertise. Social media platforms, such as Twitter and Facebook, are widely used in Saudi Arabia, with nearly 30% of Arab region Twitter users from Saudi.

## Research Gap

This research study aims to fill the gap mentioned by Yang et al. (2014) after developing the scale for measuring the innovation in blogging services. The researchers suggested that this scale must be checked for the blog reader's loyalty while keeping in mind the relationship quality as a mediating factor, which includes trust, satisfaction, and commitment. This research gap is comprehensively supported by service innovation theory, Internet service quality theory, and the commitment trust theory of relationship marketing.

As the above paragraph supports from a theoretical point of view, there are numerous examples showing that there are several websites that have indicated tremendous success in their early years. However, over time, they could not keep up with the changing trends and failed to incorporate innovations into their website services, or they failed to update those services, which resulted in lower profits, increased operational costs or, in many cases, a complete shutdown of relevant services or a website (Sadiq et al., 2020b; Alismaiel et al., 2022). The name of these websites are given below, but this phenomenon is not limited to just these websites. Furthermore, these websites are among the ones most popular globally, while thousands of sites were not very famous and simply vanished. That is why there is a need to do that kind of research to fill this gap and identify those factors that fail these well-invested and professionally supported blogging and social media platforms.

## The Rationale of the Present Study

In today's fast-paced environment, blogging services play a significant role in advising and making suggestions to target customers regarding appropriate products and services provided by different organizations according to their customized needs and wants. In this era of extremely tough competition, blog service providers aim to introduce innovations into their services to attract and retain old, as well as new blog readers. However, they do not know what kind of innovations attract their visitors in a way that they remain loyal to that specific blog service provider. This research aims to find out the most and least appropriate dimension regarding innovation in blogging services. Yang provided those innovations in 2014 in his research study about the innovation in blogging and social media website services (Yang, 2014). Moreover, this study would like to implement this instrument while keeping in mind the online relationship quality as a mediating variable and customer loyalty as a dependent variable for which the researcher also develops the model.

Previous research has focused on e-commerce innovation while failing to adequately focus on blogging services and innovations in blogging services while considering its underlying dimensions (Wu and Hisa, 2005; Liao et al., 2011). Yang identified this research gap in 2014 and subsequently developed a scale for innovation in blogging services. However, that research did not elucidate how this newly developed scale is related to visitors' loyalty while keeping in mind relationship quality as a mediating factor (Yang et al., 2014). The above discussion shows that this problem has been established. It needs to be solved from the blogger's perspective so that bloggers can focus on the dimensions that are critical from the visitor's viewpoint (Sayaf et al., 2022).

The researcher solved this problem by developing a model that uses blog service innovation as an independent variable and visitors' loyalty as an independent variable while considering the online relationship quality as a mediating factor. In this way, the author identifies which dimensions have the most and least positive influence on customer loyalty. Finally, the author suggests recommendations to bloggers about the dimensions that could ultimately reduce website development prices and different maintenance services while also increasing customer loyalty toward their blogging services.

### Research Question

1. Can we consider innovations in social media blogging services as an appropriate tool to enhance the online loyalty of website viewers toward any specific website so they could confidently record their feedback related to neighborhood management services provided by any specific organization.
2. Do all kinds of social media blogging service innovations have a positive relationship with a viewer's e-loyalty through online relationship quality toward any specific social media website?

### Research Objectives

1. The objective of this study is to provide a comprehensive solution to different organizations regarding the loyalty of their target viewers by considering a specific aspect of neighborhood management.
2. This study will also help different organizations to work on their relationship strategies with their final website viewers. In this way, they could be able to identify that on which relationship strategy they need to work from their organizational strategic perspective if they would like to get feedback on their neighborhood management services from their target respondents.

## Significance of the Study

This study's findings help different social media blogging websites to inculcate innovation in their website-related services. This research could effectively and efficiently serve their target customers and enhance their loyalty by investing in the most attractive service innovation dimension to reduce cost effectively. Furthermore, they could focus their resources and money on the aspects and innovations, which are most important and relevant to the target customer's needs and wants. Moreover, this research's conclusions would help social media blogging service providers serve their consumers better and decrease website costs by devoting themselves to the most successful and economic services innovation levels. Therefore, they must target their efforts on the most effective service type in line with the target audience's needs and demands.

## Problem Statement

There are over 600 million social media blogging websites on the world wide web today. Of these, less than 50 million are active. Despite the developing trends in technology, there are numerous websites that are inactive or forced to shut down due to less number of visitors, increased operational costs, and less profitability. The website lives on the loyalty of its existing and future viewers, but the bloggers are unable to understand a relationship with their online viewers effectively. Hence, there is a strong need to bridge this gap, resulting in the closure of several websites. The same issue is faced by the different private and governmental organizations when they would like to gather data related to neighborhood management services from their target respondents. Their social media blogging websites are outdated that they failed to attract target respondents to provide their feedback on their social media blogging websites. This problem needs immediate attention to sort out on a priority basis for enhanced connectivity and prompt problem solving related to neighborhood management services. Similar to many other countries, Saudi Arabia has been hit by the social media applications phenomenon (Alamri et al., 2020; Sayaf et al., 2021). However, there is a lack of research on social media applications use in Saudi Arabian higher education, and there is a lack of models regarding student satisfaction and academic performance, including the utilization of social media applications in Saudi Arabian higher education (Alyoussef et al., 2019; Al-Rahmi et al., 2020). Therefore, the current study attempted to minimize the literature gap by examining the use of social media applications for augmenting audiences' interaction on social media.

## Theoretical Model Development

Before moving on to the detailed literature review, it is paramount to understand why the research is carried out on these specific variables. Why are these specific variables selected as independent, mediator, and dependent variables, and what kinds of theories comprehensively support them.

As discussed earlier, the world is moving from physical business to online business, and this kind of business falls under the category of a service provider, whether you are selling any type of product online. Still, the services you are providing through your website fall under services. Every product and every service provided by an organization, whether offline or online, needs consistent improvement and innovation. They can provide better and more effective services to their target audience with enhanced organizational performance and output (Casaló et al., 2008). The primary purpose of these innovations is to develop a relationship with your target audience, and this relationship is based on some key elements, which are trust, satisfaction, and commitment (Kaplan and Nieschwietz, 2003; Carter and Bélanger, 2005).

Without building these relationships with the target customer, the organization could not develop a loyal behavior in their target customer toward the products or services which are provided by the organization (Casalo et al., 2007). Previous researchers have identified the importance of a website as a service provider, in which they discussed how websites provide different kinds of online services to cater to the needs and demands of customers online (Wang and Tang, 2003; Casalo et al., 2008; Gunadi, 2020; Nguyen et al., 2020). Moreover, they also highlighted their importance in developing a sustainable relationship with their target customer so they could be able to retain their long-term loyalty.

It is vital for any theoretical model to be based on a relevant and robust theory so the study's logic could be built on those theories. For this purpose, this research study provides two completely relevant theories in line with the theoretical framework of this study.

### The Commitment Trust Theory of Relationship Marketing

This theoretical model is based on the commitment trust theory of relationship marketing (Morgan and Hunt, 1994; Hsu et al., 2010). This theory could find its traces from previous studies which discussed the different website quality factors (Firoiu et al., 2002; Kuipers, 2002), whereas the commitment trust theory of relationship marketing explicitly discusses the relationship quality factors, including trust, satisfaction, and commitment. Considering this theory could effectively encompass the theoretical model which was discussed in this research paper. Previous research studies have also used this theory to support their model for a similar kind of academic development (Friman et al., 2002; MacMillan et al., 2005; Mukherjee and Nath, 2007; Hashim and Tan, 2015; Goutam and Gopalakrishna, 2018).

### The Expectancy Disconfirmation Theory

Further, this theoretical model is also supported by expectancy disconfirmation theory (Collier and Bienstock, 2006) which provides the basis for measuring service quality and especially internet service quality. This research encompasses the four major elements of internet service quality: efficiency, fulfillment, system availability, and privacy (Quach et al., 2016).

### Graphical Presentation of Theoretical Framework

The focus of this research study guides us toward a specific path that generates certain questions. What kind of specific services develop what kind of relationship? Without identifying these services and their impacts on relationship building, the dream of achieving customer loyalty while keeping operational costs to a minimum level is out of question. Previous studies related to this specific field have extensively used these theories to support their theoretical framework or modeling, which is why these theories also support this study's theoretical framework considering previous research findings. But to comprehensively understand this phenomenon, this research needs to present this complete concept graphically. This graph provides a better picture of the authors' concept toward the relevant problem and provides an in-depth understanding of the discussed phenomena and will guide us toward its comprehensive and applicable solution.

## Discussed Variables in the Theoretical Framework

### System Operations Related to Innovation

As this study already discussed, blog service innovation or the reader could also understand it as an innovation in blogging services consists of the innovations related to its five main dimensions, among them the first dimension is the System operation–related innovation. System operation–related innovation is based on several different components. These components are based on the humanization of a system's interface, the miniaturization of blogs, ease of use, the personalisation of services, user-friendly interactivity, and the integration of blogging platforms (Yang et al., 2014).

System operation–related innovations can be further subdivided into the humanization of system interference, which is a crucial factor in e-commerce and blogging services these days. According to several researchers (Podger, 1976; Smith, 1980; Durand and Dubreuil, 2001; Liinamaa and Gustafsson, 2010), system interference is all about recognizing the human demands and adjusting the interface of blogging services or e-commerce websites to meet those wants and expectations. There are ways to improve the user experience on blogging and e-commerce websites. We may further increase and boost productivity by combining a component connected to innovation with the simplicity of using a blogging or e-commerce website on our portable devices, which is dependent on how readily people can use certain blogs or websites (Cayzer, 2004; Boulos et al., 2006; Watson, 2011). According to Duffy and Bruns (2006), innovations in the ease of use and user-friendly interactivity in blogging services make up another significant factor in their popularity among blog readers (Farkas, 2007; Radclyffe-Thomas, 2012). The integration of blogging platforms is related to incorporating blogging functionality into an existing website. The innovation in this feature is another essential factor that makes blogging websites popular.

System operation–related innovation is based on several different components. These components are based on the humanization of the system interface, the miniaturization of blogs, ease of use, the personalisation of services, user-friendly interactivity, and the integration of blogging platforms (Yang et al., 2014). Service innovation has prevailed in literature for quite some time. However, when this study talks about service innovation in an online context, it requires much clarification from the blogger's perspective (Cayzer, 2004). Therefore, bloggers could understand this concept comprehensively to enhance their websites' performance and users' experiences.

### Humanisation of System Interface

Understanding innovations related to system operations is a daunting task. The concept of system operation–related innovation was proposed by Yang et al. (2014). This study already discusses that service innovation is a relatively new concept, and understanding it from an online perspective is an entirely new task (Berry et al., 2006). The first approach to understanding the innovations related to system operations is the humanization of the system interface. To understand it, the author needs to divide it into two parts: humanization and the system interface. Humanisation is the process of changing or converting the procedure of interaction between humans and machines so that the human mind can understand it more appropriately and clearly (Beck and Prügl, 2018).

The word “humanization” is seen from different perspectives by different scholars. To understand it from the user's perspective, it must be kept in mind that the user is using some kind of machine or interface that needs to be developed so that it feels more human in its interaction with end-users. Hsu and Lin (2008) further clarify humanisation's connection with system interface in such a way that the bloggers must develop the website's functions or complete functionality by keeping in mind the human and end-users' requirements.

The other part of this approach is the system interface. Hookway (2014) says that computing an interface is a connection between two or more computer systems that share data in various ways. There may be a transfer of information between software, computer hardware, peripheral devices, or even people.

While considering innovations in services from a system operation's perspective, which are specifically related to blogging or social media website's point of view, organizations need to introduce innovations in the humanization of system interface. In this way, the user can interact with the system in a more synchronized and harmonious manner. These considerations have enhanced performance and increased user loyalty to blogging and social media websites (Farkas, 2007).

### Miniaturization of Blogs

A miniaturization of blogs approach is presented by Yang et al. (2014) from the perspective of innovation in services that are primarily related to system operations. To understand this concept, the author again needed to divide this approach into two main concepts: miniaturization and blogs. For a better understanding, this study first focused on what blogging is.

Blogging is an Internet-based second-generation activity that is extremely popular these days for its effective communication and domino effect regarding different services (Ip and Wagner, 2008). Johnson and Kaye (2004) said that blogging is a web-based diary-style activity that provides observations, recommendations, and suggestions regarding different products and services in combination with commentaries and recommended links for different useful articles and videos. Li and Chen (2009) further clarify the concept of blogging by saying that blogs serve as a model of an online social network, which represents frequently updated web-based commentaries with reverse sequential sequences of dated entries, which could be checked and read by the blog browsers at any time.

Comprehending this idea within the context of intangible items or services, such as blogging or social media websites, is another task entirely. It could be summarized as a service provided by the blogger or social media website to automatically adjust the screen resolution of their websites so that it is equally responsive on mini-screens, such as those of mobile phones or tablets. Hence, blogs' miniaturization ultimately provides more ease of use and better user engagement on small screens.

### User-Friendly Interactivity

Innovations linked to services are always related to the interactivity between a customer and a service provider to generate a positive and friendly atmosphere that fosters long-lasting relationships (Motta et al., 2018). Understanding this phenomenon ultimately concludes that user-friendly interactivity is a vital part of service innovation (Ke et al., 2012). Either this innovation is in a traditional service sector environment or an online blogging or social media website environment. However, to comprehend interactivity from an online perspective, we need to explore this approach profoundly. Preece and Rombach (1994) say that in computers, interactivity is the dialogue that occurs between human beings through an interface or between a human being and a computer program. Another example of online interactivity is products and services on digital computer-based systems that respond to the actions of the user by displaying content, such as text, moving images, animation, video, audio, or video games, to solve problems or queries provided by a human user on the other end. Online interactivity (Kieras and Meyer, 1997).

### Defining Ease of Use

The primary purpose of providing innovation in services is to promote and provide ease of use for the target customer and viewer toward the usage of that particular service. This way, they could come back again, ultimately enhancing the relationship between customers and organization resulting in more profitability (Yang et al., 2014). The organizations should always aim to understand the concept of “ease of use” from the product's or service's point of view. Furthermore, providing innovations in a tangible product with an intention to make it more user friendly is always a priority for manufacturers (Motta et al., 2018). However, providing innovation regarding ease of use of services can get confusing. That is why the author could define the concept of innovation in services from the ease of use perspective as follows: it is an effort from service providers to provide services in such a way that users could be able to use those services in a more relaxed and understandable way (Ke et al., 2012). The provided services must not be presented in such a way that they create a different kind of hurdle in the process of just using them, and when the author discusses this matter from an online perspective, this concept presents itself in a more challenging way (Khan et al., 2015).

### Personalisation of Service

This invention also contributes significantly to the growth of consumer loyalty for your blogging or social media website. Personalisation is critical for building a strong and loyal client database (Bettencourt and Ulwick, 2008). Personalisation can be a means of meeting the customer's needs more effectively and efficiently, thus making their interactions faster and easier, consequently increasing customer satisfaction and the likelihood of repeat visits. The concept of product personalisation could be defined as alternation in the product according to the customer's needs or desires (Leão and Mello, 2007). Using product personalisation software, it can comprise custom-made products or specific designs/prints on already available products. When the author applies this concept to the personalisation of services, especially from an online perspective, it gets even more specific and customer oriented. Users want different blogging and social media websites to provide services according to their interests, which can be identified through their browsing history (Srinivasan et al., 2002).

### Integration of Blogging Platforms

This type of innovation is specifically related to an online perspective. The researchers could define this kind of innovation in such a way that there is a need to integrate different blogging and social media platforms for effective and efficient engagement between viewers (Liinamaa and Gustafsson, 2010). Users use different kinds of blogging and social media platforms to express their ideas and views, and they are in dire need to represent them on one platform and simultaneously broadcast them on other platforms. Therefore, innovations related to services in connection with the integration of different blogging platforms have received complimentary views from online users (Radclyffe-Thomas, 2012).

### Related Hypotheses

H^1^: System operation–related innovations incorporated by blogging and social media websites have a positive impact on Website Viewer's e-loyalty.

This relationship is supported by the famous “commitment trust theory of relationship marketing.” System operation is the dimension of the blog service innovation, which is why this theory is equally applicable to blog service innovation as it is to system operation. Moreover, it is also necessary to determine whether that specific innovation has any kind of relationship with said variables.

H^2^: System operation–related innovations incorporated by blogging and social media websites have a positive impact on Website Viewer's e-loyalty under the mediation of satisfaction.

This relationship is also supported by both the “expectancy disconfirmation theory” (Collier and Bienstock, 2006) and the “commitment trust theory of relationship marketing.” Furthermore, we need to understand whether satisfaction could play its role between independent and dependent variables. So, this study could understand and find its implications on organizational productivity for better performance and effective cost minimization.

H^3^: System operation–related innovations incorporated by blogging and social media websites have a positive impact on the Website Viewer's e-loyalty under the mediation of commitment.

This relationship is also supported by both the “expectancy disconfirmation theory” (Collier and Bienstock, 2006) and the “commitment trust theory of relationship marketing.” Furthermore, we need to understand whether commitment could play its role between independent and dependent variables. So, this study could understand and find its implications on organizational productivity for better performance and effective cost minimization.

### Social Technology–Related Innovations

Innovations related to social technology–related innovation in blogging websites forms another significant factor. According to Yang et al. (2014), the social technology developments are based on, but not limited to, the social services included in the blogging website. How much technology compatibility do the blogging websites have? Finally, the blogging website should have a microblogging and co-broadcasting capability. Previous research indicates (Keenan and Shiri, 2009) that the chance of social interactions provided by blogging websites among different people and communities is an essential factor in their popularity. Blog readers like to interact with other people easily and collaboratively (Freeman, 2004). It is also said that with every passing day, the introduction of new technologies in the field of e-commerce and blogging has made it necessary to incorporate the feature of new technology compatibility on websites (Hernández Ortega et al., 2007). This way, they could be able to provide their blog readers and users with more up-to-date technology features, environment, and services for enhanced productivity and performance (Hernández Ortega et al., 2007). In collaboration with the above-mentioned components of social technology–related innovation, it is also essential to understand the function of co-broadcasting and microblogging on the blogging websites, as clarified in previous works (Coeckelbergh, 2011; Liu et al., 2012; Zhang and Peng, 2015). This demonstrates that microblogging is a feature available on blogging websites that enables the interchange of little contents, such as video links, individual photographs, or brief words; blogging websites must also be capable of broadcasting these small contents across many platforms (Huo et al., 2021).

### Social Function

This is defined as the ability of the website to provide the facility for interaction between users (Weaver and Morrison, 2008). The interaction could be explained in different situations. It could be the interaction *via* comments on a post, picture or video, personal chat, or *via* group chat. A personal e-mail or group email facility could also be considered a social interaction. Different social functions are considered as ways to share one's social activities online *via* picture or video posting or by sharing one's feelings online, which could effectively connect you with your family and friends and make you alive online (Keenan and Shiri, 2009).

### Technology Compatibility

This is defined as the website's ability to be compatible with all the latest social platforms and technologies. This compatibility includes but is not limited to the opening of websites in different browsers and platforms, consisting of Windows, Mac, and Linux operating systems. Furthermore, over time, other mobile phone companies have introduced their operating systems, which are primarily based on Android but are still different from one another (Azim et al., 2016).

### Co-Broadcasting Function

This is defined as the ability of the website to provide the facility to share your ideas with other social platforms at the same time (Ormseth and Quirino, 2018). Over time, there are so many social interaction websites and applications that user requires to post on one website, and then it provides the facility of sharing that post on other social media applications simultaneously.

### Related Hypotheses

H^4^: Social technology–related innovations incorporated by blogging and social media websites have a positive impact on Website Viewer's e-loyalty.

This relationship is supported by the commitment trust theory of relationship marketing and the expectancy disconfirmation theory. Moreover, it is also necessary to determine whether that specific innovation has any kind of relationship with said variables.

H^5^: Social technology–related innovations incorporated by blogging and social media websites have a positive impact on Website Viewer's e-loyalty under the mediation of satisfaction.

This theory is supported by the commitment trust theory of relationship marketing and the expectancy disconfirmation theory. Furthermore, we need to understand whether satisfaction could play its role between independent and dependent variables. So, this study could understand and find its implications on organizational productivity for better performance and effective cost minimization.

H^6^: Social technology–related innovations incorporated by blogging and social media websites have a positive impact on the Website Viewer's e-loyalty under the mediation of commitment.

This theory is supported by the commitment trust theory of relationship marketing and expectancy disconfirmation theory. Furthermore, we need to understand whether commitment could play its role between independent and dependent variables. So, this study could understand and find its implications on organizational productivity for better performance and effective cost minimization.

### Satisfaction

Satisfaction is a hot issue these days, and academic scholars and marketing professionals alike have developed a growing interest in this idea over time (Nusair and Kandampully, 2008). Oliver (1981) introduced an expectation disconfirmation model that is widely used to analyse customer satisfaction levels in the service and retail sectors. It indicates that customers evaluate the performance of products or services against their expectations for those products or services, which can result in satisfaction if performance exceeds expectations, or in dissatisfaction if expectations are greater than the performance of the organization's or blogging website's product or service (Hennig-Thurau et al., 2002).

However, with the advancement of technology and the transfer of business from a conventional to an online setting, it is becoming more difficult for a client to discern if the organization's services perform better or worse than their intended expectations (Zeithaml, 2000; Allagui and Temessek, 2004). Rust et al. (1999) argued that when our target customer's expectations are unclear, these businesses must compute our consumers' perceptions about our product or service, which are presently based on their experiences with the organization's services.

H^7^: Satisfaction has a positive impact on Website Viewer's e-loyalty.

**Figure 1:**
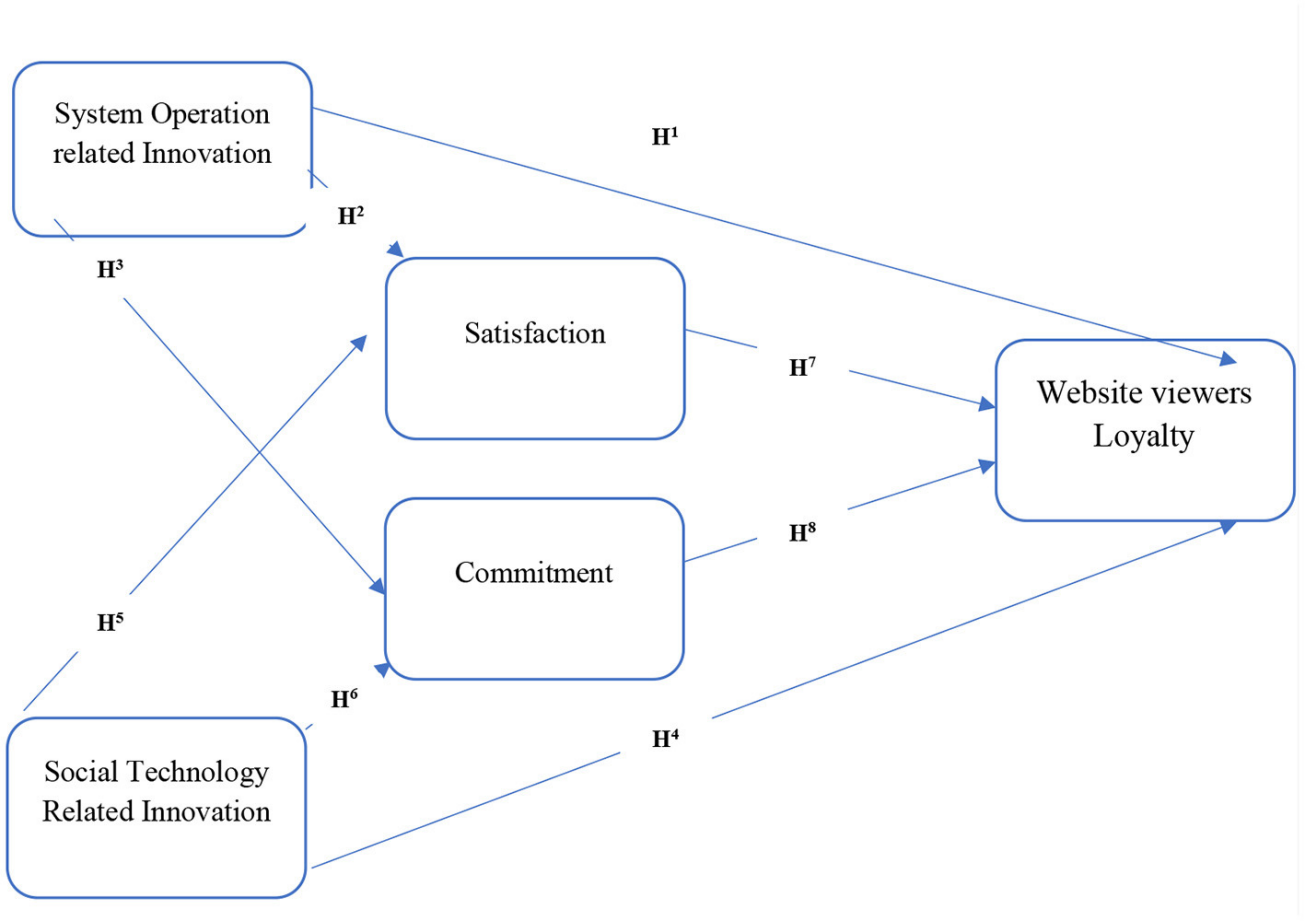
Theoretical framework.

This relationship is supported by the commitment trust theory of relationship marketing and the expectancy disconfirmation theory. Furthermore, it is also needed to understand whether online relationship quality could play its role between independent and dependent variables. So, this study could be able to understand and find its implications on organizational productivity for better performance and effective cost minimization. Figure 1 shows the graphical representation of hypotheses.

### Commitment

Commitment is considered one of the key concepts in relationship marketing (Keh and Xie, 2009). It is also believed that marketing scholars have borrowed this concept from the literature of organizational behavior, and commitment is genuinely originated from the theory of social exchange (Cook and Emerson, 1978; Fullerton, 2005). Although there are numerous definitions of commitment available in the literature, one of the most suitable definitions of commitment to today's online business environment is presented by Liang and Chen (2009): it is “the consumer's psychological attachment toward the online service provider, along with his/her willingness to maintain the customer–firm relationship.” This concept is further strengthened by other researchers (Morgan and Hunt, 1994; Eastlick et al., 2006; Theron and Terblanche, 2010) who state that Commitment grows when consumers or customers are willing to prepare the work, make significant efforts to enhance and maintain the relationship with the particular firm and invest significant resources to improve the affiliation between the organizations and themselves (Abdullah et al., 2021b).

H^8^: Commitment has a positive impact on Website Viewer's e-loyalty.

This relationship is supported by the commitment trust theory of relationship marketing and the expectancy disconfirmation theory. Furthermore, we need to understand whether online relationship quality could play its role between independent and dependent variables. So, this study could understand and find its implications on organizational productivity for better performance and effective cost minimization.

### Website Viewer's e-Loyalty

E-loyalty is “a favorable attitude of the consumer toward an electronic enterprise leading to repeated buying behavior.” It comprises excellent customer service, prompt delivery, enticing product submissions, easy and fairly priced shipping, and handling. E-loyalty is described as the “positive consumer approach to an electronic business, shown by recurrent buying activity.” Superior customer service, prompt delivery, interesting product presentation, simple and cost-effective shipping, and handling, as well as clear and reliable privacy policies, are included. Consequently, it is essential to examine the history of e-loyalty. Satisfaction, confidence, quality of service, and perceived value are just a few examples (Sadiq et al., 2020a). The development and maintenance of customer loyalty and satisfaction is thus a key objective for online companies to increase profitability, acquire and maintain a competitive advantage, and implement clear and reliable privacy rules. Consequently, the analysis of the history of e-loyalty has become an important precedent for satisfaction, trust, quality of service, and perceived value (Li et al., 2021).

The major goal of building client loyalty and satisfaction thus is to enhance profitability and to gain a competitive edge for online businesses. With the recent spread of COVID-19, the world is quickly changing from a physical to an online paradigm. Everything is forced to shut down or shift its operations online (Addo et al., 2020). Online business is the future of the world economy (Sheng and Liu, 2010). Whether it is a business or education sector, if you want to run it, you have to find out how you could continue your operations online. Otherwise, your business or enterprise's survival could be jeopardized due to the pandemic situation (He and Harris, 2020). Considering this situation, everybody wants to secure the loyalty of their online customer, so they could be able to earn profits or even sustain their business in this situation. Therefore, the company's online presence and its future sustainability with the database of loyal customers is a vital factor for online survival (Abdullah et al., 2021a).

## Research Methodology

In this study, the information of the respondents was collected from the admission office, or the class attendance register of different educational institutes to get the targeted response rate. The data were collected with the help of friends and randomly from different educational institutes in Pakistan and Saudi Arabia.

### Data Analysis

Data analysis was performed using the Statistical Package for the Social Sciences (SPSS 25) and AMOS 24 software packages. The data were collected from almost 350 students who belong to the management sciences and computer sciences departments of different universities in Pakistan and art education students at King Saud University in Saudi Arabia. Among these 500 respondents, the researcher received 287 completely filled questionnaires, hence the response rate of 57.4% was calculated for this research study. The purposive sampling methodology is used to gather data from the respondents.

The reliability analysis in Table 1 is shown. In this research, each variable has a satisfactory reliability alpha value.

**Table 1:** Reliability analysis of all variables.

**Variables**	**Items**	**Cronbach's Alpha value**
System operation related Innovation	6	0.805
Social technology related Innovation	3	0.754
Satisfaction	5	0.800
Commitment	5	0.744
Website viewer's loyalty	3	0.836

### Instrument

For measuring system operation–related innovation and service privacy–related innovation, the proven scale is used which is developed by Yang (2014) and it employs a five-point Likert scale ranging from strongly agree to strongly disagree; it is substantially changed and changed to meet the study's objectives and is further validated by CFA analysis. To measure the concept of satisfaction and commitment, we used the scale developed by Brun et al. (2014). While for measuring consumers, we used the scale which developed by Allagui and Temessek (2004) and we slightly modified it to fit the scope of this study and then verified by the CFA methodology.

### Confirmatory Factor Analysis

Pooled CFA is a latest and more reliable technique. In this methodology, the AMOS 24 runs all latent variables simultaneously (Afthanorhan et al., 2014; Chong et al., 2014). Table 2 shows the CFA model fitness.

**Table 2:** Pooled CFA model fitness tests.

**Name of category**	**Name of index**	**Index full name**	**Value in analysis**	**Acceptable value**	**Literature**
Absolute fit	RMSEA	Root mean square of error approximation	0.047	<0.06	(Browne and Cudeck, 1993)
Incremental fit	CFI	Comparative fit index	0.934	>0.90	(Bentler, 1990)
Parsimonious fit	Chi-sq/df	Chi-square/degrees of freedom	1.560	<3	(Hu and Bentler, 1999)

Table 3 shows the reliability value or factor loading of every item separately. It also shows the composite reliability of a complete scale of any variable. The reliability of the measurement scales was measured with composite reliability, which is preferred to report a scale's reliability (Netemeyer et al., 2003), a widely used indicator.

**Table 3:** Pooled confirmatory factor analysis.

**Independent variables**			
How much important it is from your point of view that social media websites and other blogging websites need to provide “INNOVATION” in the below-mentioned “SERVICES” which are provided by them to their online users while keeping in mind the neighborhood management.			
**Scale**	**Items**	**Factor loadings**	**Scale reliability**
System-operation-related innovation	Miniaturization of blogs.	0.75	0.805
	Humanisation of system interference.	0.60	
	User-friendly interactivity.	0.80	
	Ease of use.	0.71	
	Personalisation of service.	0.82	
	Integration of blogging platforms.	0.80	
Social technology-related innovation	Social function.	0.78	0.754
	Technology compatibility.	0.88	
	Co-broadcasting function.	0.76	
**Mediating variables**			
Keep any Social Media or Blogging website (Facebook, Twitter, Linked-In, etc) in your mind (Considering neighborhood management services) that you frequently use while answering below mentioned questions.			
**Scale**	**Items**	**Factor loadings**	**Scale reliability**
Satisfaction	I am very satisfied with the ease of use of this web site.	0.72	0.800
	I am very satisfied with the information provided by this web site.	0.81	
	I am very satisfied with the personalisation offered by this Website.	0.89	
	My experience with this web site is very satisfactory.	0.75	
	I am very satisfied with the design of this web site.	0.83	
Commitment	I really like this web site.	0.60	0.744
	I am very attached to this website.	0.88	
	I feel a strong sense of belonging to this website.	0.73	
	It would be very difficult for me to stop using this web site.	0.65	
	I have too few alternatives to consider leaving this website.	0.86	
**Dependent variable**			
Keep any Social Media or Blogging website (Facebook, Twitter, Linked-In, etc) in your mind which you frequently use while answering below mentioned questions.			
**Scale**	**Items**	**Factor loadings**	**Scale reliability**
E-loyalty	I try to use this website whenever I need to use it.	0.87	0.836
	I like using this website.	0.89	
	I believe that this is my favorite website.	0.75	

### Assessment of Discriminant Validity

Convergent validity is a subtype of construct validity defined as follows: The term “construct validity” refers to the fact that a test is meant to assess a certain construct. Convergent validity is the ability to demonstrate that two measurements that are meant to assess the same concept are assessing the same phenomena. On the other hand, discriminant validity indicates that two measurements that are not meant to be associated are not associated. Excellent construct validity requires the presence of both forms of validity. The HTMT analysis was used to determine discriminant validity, with the cut-off threshold for severe discriminant validity being 0.850 and for liberal discriminant validity being 0.900 (Henseler et al., 2015). The values shown in Table 4 demonstrate that the items fulfill the discriminant validity criteria.

**Table 4:** HTMT analysis.

	**System Operation related innovation**	**Social Technology related innovation**	**Satisfaction**	**Commitment**	**Website viewer's loyalty**
System operation related innovation	X				
Social technology related Innovation	0.194	X			
Satisfaction	0.284	0.070	x		
Commitment	0.264	0.041	0.500	x	
Website viewer's loyalty	0.071	0.001	0.011	0.080	x

### Path Analysis in Structural Equation Modeling

The postulated relationships are investigated in this work using structural equation modeling (SEM). This analysis includes exogenous factors to facilitate the examination of endogenous variables through AMOS 24. In this study, you can see that independent and dependent variables are linearly related to one another. The basic design was constructed by using observed facts to build on. All observations were tabulated and linked to information on their mean values for analysis. Table 5 shows the model fit indices for the structural model, and it shows that they are meeting the acceptance criteria.

**Table 5:** Structural equation modeling – model fitness test.

**Name of category**	**Name of index**	**Index full name**	**Value in analysis**	**Acceptable value**	**Literature**
Absolute fit	RMSEA	Root mean square of error approximation	0.053	<0.06	(Browne and Cudeck, 1993)
Incremental fit	CFI	Comparative fit index	0.916	>0.90	(Bentler, 1990)
Parsimonious fit	Chi-sq/df	Chi square/degrees of freedom	1.576	<3	(Hu and Bentler, 1999)

Table 6 illustrates the independent variable's direct influence on the dependent variable.

**Table 6:** Results of direct effects in structural model.

**Hypothesis**	**Causal path**	***P*** **-value**	**Standardized estimated**
H^1^	System Operation related Innovation → Website viewer's loyalty	0.793	0.022
H^4^	Social Technology related Innovation → Website viewer's loyalty	0.242	0.068
H^7^	Satisfaction → Website viewer's loyalty	0.001	0.195
H^8^	Commitment → Website viewer's loyalty	0.001	0.378

Table 7 shows that all the hypotheses are statistically significant, except H^3^. The *p* of all other hypotheses is <0.05 which shows that the confidence interval is 95%. Also, Table 7 shows the path analysis of structural equation modeling.

**Table 7:** Results of in-direct effects in structural model.

**S/R**	**Hypothesis**	**Direct beta without mediation**	**Direct beta with mediation**	**Indirect beta/standardized estimates**	**Mediation type observed**
H^2^	System Operation related Innovation → Satisfaction → Website viewer's loyalty	0.022	−0.005	0.059^***^	Full mediation
H^3^	System Operation related Innovation → Commitment → Website viewer's loyalty	0.022	0.062	0.044	No mediation
H^5^	Social Technology related Innovation → Satisfaction → Website viewer's loyalty	0.068	0.090	0.03^**^	Full mediation
H^6^	Social Technology related Innovation → Commitment → Website viewer's loyalty	0.068	0.078	0.06^**^	Full mediation

*** *Significance level at 0.001*.

** *Significance level at 0.05*.

According to Table 7, three hypotheses are statistically significant, and the observed mediation for these hypotheses is classified as full mediation. Full mediation implies that the mediating variable explains the whole link between the independent and dependent variables, while partial mediation implies that the mediating variable explains some, but not all, of the link between the independent and dependent variables. Only one H^3^ hypothesis is not statistically significant.

## Discussion on Results

It is evident over time that web service innovation in web service will significantly impact our lives. System operation–related innovation and social technology–related innovation are not statistically significant in a direct relationship. It is also evident from the research conducted by different researchers that innovation in webservices could not be understandably connected with the viewers' loyalty directly (Ferris and Farrell, 2003). Moreover, we must understand whether they could successfully generate loyalty through satisfaction and commitment or not (Roy and Ramanujan, 2001). Considering the results of this research work, it is evident that social technology–related innovation could be considered the most viable option to increase the loyalty of the website viewers. If any organization does not have the resources to build the webservice innovation related to social technology, they could move toward the system operation–related innovation. By using this specific innovation, the organization could increase the loyalty of their target customer through the enhancement of satisfaction. Suppose any organizations want to develop their viewers' loyalty through commitment, it is strongly recommended that they must not use the system operation–related innovation because this innovation is not statistically significant.

## Managerial Implications

In today's business scenario, when every business is shifting from an online to offline perspective or at least trying their level best to maintain their presence online, the way to tackle and attract your target customer also changes. Neighborhood management through social media could be considered a daunting task, but with the right set of information, gathering information through this medium could be possible without any hindrance. Managers of the different organizations and officials of the different government agencies are recommended to inculcate social technology–related innovation in their webservices if they want to attract their target respondents and if they want them to provide data related to neighborhood management. Improvement in neighborhood management services through social media could only be possible if the respondents are confident enough to provide their feedback on designated social media platforms (Akram et al., 2018a). Their confidence and loyalty could only boost by introducing innovations in web-related services which are social technology—and system operation—related innovations. Suppose the target of the firm or organization is to enhance the loyalty of their target respondents through the commitment factor, it is recommended that they must not use system operation–related innovation in their web services. Because using that kind of web service will not significantly impact the target's respondents' loyalty toward that specific online social media platform (Sadiq et al., 2020b).

## Research Limitation and Future Research Directions

Time and money are the two main limitations of this research work. Moreover, respondents' knowledge of neighborhood management services could also be considered the main limitations of this research work. No doubt the respondents know about the community-related and neighborhood management services, but considering the scenario of social media, providing suggestions and feedback through any specific social media platform is a new idea for them. Hence it created a significant understanding gap. Future research could focus on developing a more appropriate questionnaire related to neighborhood management services through social media.

## Data Availability Statement

The datasets presented in this article are not readily available because of ethical restrictions. Requests to access the datasets should be directed to the corresponding author, waqas_sadiq2011@hotmail.com.

## Ethics Statement

Ethical review and approval was not required for the study on human participants in accordance with the local legislation and institutional requirements. Written informed consent from the patients/participants legal guardian/next of kin was not required to participate in this study in accordance with the national legislation and the institutional requirements.

## Author Contributions

MS, CH, WA-R, AA, NA, QA-m, and SZ: conceptualization. MS, NA, WA-R, AA, and SZ: data curation and formal analysis. MS, CH, NA, and WA-R: methodology and supervision. CH, WA-R, QA-m, AA, and NA: validation. MS, SZ, WA-R, CH, and AA: writing—original draft. MS, CH, WA-R, QA-m, AA, SZ, and NA: writing—review and editing. All authors contributed to the article and approved the submitted version.

## Funding

This work was supported by the King Saud University, Riyadh, Saudi Arabia, through Researchers Supporting Project RSP- 2022/R417.

## Conflict of Interest

The authors declare that the research was conducted in the absence of any commercial or financial relationships that could be construed as a potential conflict of interest.

## Publisher's Note

All claims expressed in this article are solely those of the authors and do not necessarily represent those of their affiliated organizations, or those of the publisher, the editors and the reviewers. Any product that may be evaluated in this article, or claim that may be made by its manufacturer, is not guaranteed or endorsed by the publisher.
